# The Effect of MicroRNA 21 and MicroRNA 200b Expression on Carcinogenesis in Endometriosis-Associated Ovarian Cancers and Relationship with Clinicopathological Parameters

**DOI:** 10.3390/medicina61061035

**Published:** 2025-06-04

**Authors:** Esra Canan Kelten Talu, Emine Çağnur Ulukuş, Yasemin Çakır, Merih Güray Durak, Zeynep Bayramoğlu, Hikmet Tunç Timur, Sefa Kurt, Sefai Merve Özdemir, Safiye Aktaş

**Affiliations:** 1 Department of Pathology, Tepecik Training and Research Hospital, Izmir Faculty of Medicine, University of Health Sciences, Izmir 35540, Türkiye; 2 Department of Molecular Pathology, Faculty of Medicine, Institute of Health Sciences, DokuzEylul University, Izmir 35410, Türkiye; 3 Department of Pathology, Memorial Ataşehir Hospital, Istanbul 34750, Türkiye; cagnur.ulukus@gmail.com; 4 Department of Pathology, Faculty of Medicine, DokuzEylul University, Izmir 35340, Türkiye; pth.yasemincakir@gmail.com (Y.Ç.);; 5 Department of Obstetrics and Gynaecology, Faculty of Medicine, DokuzEylul University, Izmir 35340, Türkiye; 6 Department of Basic Oncology, Institute of Oncology, DokuzEylul University, Izmir 35330, Türkiye

**Keywords:** endometriosis, endometriosis-associated ovarian cancer, biomarker, miRNA 200b, miRNA 21, clinicopathological findings

## Abstract

(1) *Background and Objectives:* Endometriosis is defined as the presence of endometrial glands and stroma outside the uterine cavity. It affects 5–15% of women of reproductive age. Ovarian cancer develops in approximately 1% of patients with endometriosis. Prediction of those with endometriosis who will develop ovarian cancer is among the current research topics. (2) *Materials and Methods:* With this study, we aimed to reveal the role of miRNA 200b and miRNA 21 in endometriosis-associated ovarian carcinoma (EAOC). Thirteen patients diagnosed as having EAOC between 2015 and 2023 were included, with their endometriosis and eutopic endometrium tissues (Group 3: 13 patients, 39 tissue samples). Two separate groups were then detected to compare with these cases: Group 2 composed of tuba-ovarian endometriosis with its eutopic endometrium (10 patients, 20 tissue samples) and Group 1 composed of eutopic endometrium only (10 patients, 10 tissue samples). The foci marked on H&E sections were determined from the area on the relevant paraffin blocks and small tissue samples were taken in tubes to be studied with real-time PCR. (3) *Results:* No significant difference was detected for miRNA 21 and miRNA 200b expression levels among eutopic endometrium, endometriosis, and cancer foci in Group 3. However, miRNA 21 and miRNA 200b expression levels in the eutopic endometrial tissue of cases with ovarian cancer were significantly higher than in the eutopic endometrial tissues of cases with (Group 2) and without endometriosis (Group 1). (4) *Conclusions:* This study suggests that increased miRNA 200b and miRNA 21 expression levels detected in eutopic endometrial tissue of patients with endometriosis may contribute to identifying cases that may develop EAOC.

## 1. Introduction

Endometriosis is defined as the presence of endometrial glands and stromal tissue outside the uterine corpus. Affecting 5–15% of women of reproductive age, endometriosis can significantly reduce their quality of life by causing various clinical problems, such as chronic pelvic pain, dysmenorrhea, and infertility [1]. Endometriotic foci are found in the pelvic area in 2–30% of cases, while less than 4% are localized in extrapelvic regions [2]. The most common location in the pelvic area is the ovaries.

Approximately 1% of endometriosis cases develop ovarian tumors. The most frequently encountered ovarian tumors include endometrioid carcinoma and clear cell carcinoma [3]. The frequency of these two carcinomas may vary between Western countries and East Asian countries. Other ovarian tumors that may develop on the basis of endometriosis include seromucinous tumors, adenosarcoma, carcinosarcoma, and endometrial stromal sarcoma [2,3].

Currently, the gold standard for the diagnosis of endometriosis remains invasive surgical procedures and histopathological sampling [4]. Therefore, there is a need for new biomarkers that are easy to measure and prevent the use of unnecessary invasive methods to diagnose endometriosis and assess treatment response. For this purpose, many molecules with their serum and/or plasma levels have been investigated [5]. Among these molecules, the most commonly used in routine practice is Ca125, a glycoprotein. Various microRNAs (miRNAs) have been studied for their serum/plasma levels in cases of endometriosis, and it has been reported that their levels are different from normal endometrial tissue [1].

MicroRNAs are single-stranded RNA molecules, 21–23 nucleotides in length, transcribed from DNA but not translated into proteins (non-coding). They were first discovered in 1993 by Lee and colleagues during their investigation of the development of *Caenorhabditis elegans* nematodes at the Ambros lab, and the term “microRNA” was coined in 2001. The primary role of these small molecules is to regulate gene expression post-transcriptionally by influencing messenger RNAs (mRNAs) [1]. A single miRNA can affect multiple mRNAs, and multiple miRNAs can also influence the same mRNA. miRNAs exert their essential effects on the process of carcinogenesis by influencing the expression levels of oncogenes and tumor suppressor genes [6,7]. At this point, some miRNAs show more oncogenic effects (such as miR-21 and miR-155), while others exhibit more tumor suppressor effects (such as miR-200 and miR-let 7 families) [6,7]. Furthermore, it is known that miRNAs are involved in many stages of carcinogenesis, including insensitivity to growth-inhibitory signals, self-sufficiency in growth signals, evasion of apoptosis, limitless cell proliferation, invasion, metastasis, angiogenesis, and genomic instability [7].

The relationship of miRNAs with carcinogenesis, their tissue- and tumor-specific expression levels, their resistance to changing environmental conditions, stability, ability to pass into plasma and serum, and detection through minimally invasive procedures make them favorable as biomarkers [1,8]. An ideal biomarker is expected to be useful for the early diagnosis of cancer, treatment, and/or prognosis, detectable through non-invasive methods, have a low cost/performance ratio, provide consistent results in repeated measurements, and demonstrate high sensitivity and specificity.

When ovarian cancers cause clinical symptoms, they are usually in advanced stages. Therefore, the identification of biomarkers that assist in the early diagnosis of ovarian cancer has gained importance. In this study, we aimed to reveal the role of miRNA 200b and miRNA 21 in EAOC.

## 2. Materials and Methods

### 2.1. Study Design and Patient Selection

This retrospective cross-sectional study includes 3 groups of patients. By scanning the hospital computer system, all patients who were operated between 2015 and 2023 and diagnosed with endometriosis and ovarian carcinoma accompanying endometriosis in their excision materials were identified and constituted Group 3. Patients who were operated in the same year range, diagnosed with endometriosis in excision materials, without ovarian cancer or any other type of cancer in their clinical follow-ups constituted Group 2. The normal endometrial tissues of excision materials of women who had undergone surgery for various reasons, such as leiomyoma uteri, without the diagnosis of endometriosis, were also identified for comparison with the previous two groups, and these patients constituted Group 1. This study was approved by the Dokuz Eylül University Non-Interventional Research Ethics Committee on 11.09.2024 with the decision number 2024/30-54.

A clinical sample size calculation program was used to determine the minimum number of samples for adequate sample power. Study group design was set according to independent groups and continuous mean primary endpoints. “Significance” was set as 0.05 and “power” was at 90%. According to anticipated means and standard deviations of the groups, the minimum sample number required for each group was 7.

### 2.2. Histopathological Evaluation and Tissue Sampling Methods

Hematoxylin–eosin (H&E)-stained tissue sections of all cases were re-evaluated under a light microscope by an expert pathologist, and suitable tissue areas for Groups 1, 2, and 3 were determined in H&E sections. Accordingly, endometrium with normal morphology (eutopic endometrium) in Group 1, eutopic endometrium and foci of endometriosis in Group 2, and eutopic endometrium, foci of endometriosis, and ovarian cancer in Group 3 patients were marked on paraffin blocks, and small pieces of tissue were taken in tubes for analysis. In the histopathological evaluation, cases that did not contain endometrial tissue or were shed, as well as cases that contained only basal endometrium or additional lesions (such as endometrial polyps, etc.), were excluded from the study. Tumor sampling was performed from areas rich in viable tumor cells and devoid of necrosis and hemorrhage.

### 2.3. RNA Isolation and cDNA Synthesis

Total RNA was isolated from formalin-fixed, paraffin-embedded (FFPE) tissue samples using the Norgen Biotek FFPE RNA Purification Kit (Thorold, ON, Canada). Briefly, after deparaffinization, tissue samples were lysed, and RNA was bound to columns, treated with DNase I, washed, and eluted.

MicroRNA cDNA synthesis was performed using the Norgen Biotek MicroScript microRNA cDNA Synthesis Kit (Thorold, ON, Canada) according to the manufacturer’s protocol. The reaction was carried out at 37 °C for 30 min, 50 °C for 30 min, and 70 °C for 15 min.

### 2.4. Quantitative Real-Time PCR Analysis

The expression levels of miR-21 and miR-200b were analyzed using FastStart Essential DNA Green Master mix (Mannheim, Germany) QPCR included an initial denaturation at 95 °C, followed by annealing at 53 °C, and extension at 72 °C in a standard three-step amplification protocol. QPCR results were evaluated by Roche LightCycler Nanosystem program. Quantiative miR-21 and miR-200b levels were normalized to the expression of U6. Primer sequences were as follows: miR-21 forward primer: 5′-GCTTATCAGACTGATGTTG-3′ and reverse primer: 5′-GAACATGTCTGCGTATCTC-3′; miR-200b forward primer: 5′-CTTACTGGGCAGCATTG-3′ and reverse primer: 5′-GAACATGTCTGCGTATCTC-3′ U6 forward primer: 5′-CTCGCTTCGGCAGCACAT-3′ and reverse primer: 5′-TTTGCGTGTCATCCTTGCG-3′.

### 2.5. Statistical Analysis

Differences in miRNA expression levels between the groups were evaluated using the Mann–Whitney U test by the IBM SPSS 29.0 program. *p* value < 0.05 was considered statistically significant. Expression levels of miRNAs of more than 600 were normalized to 600.

## 3. Results

### 3.1. Clinicopathological Findings

**Figure 1:**
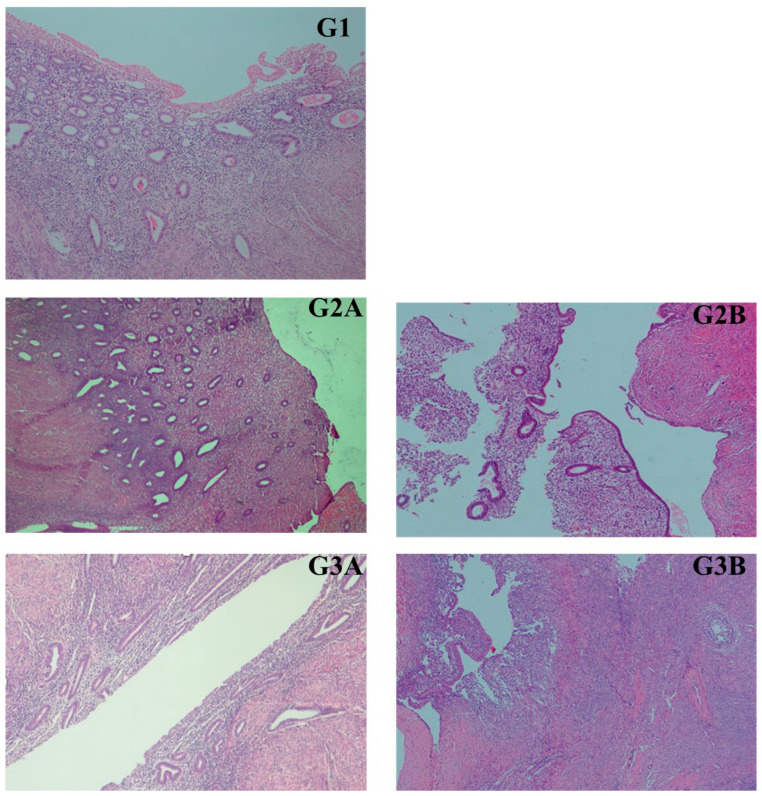
Group 1: eutopic endometrial tissues of Group 1; Group 2A: eutopic endometrial tissues of Group 2; Group 2B: endometriotic tissue of Group 2; Group 3A: eutopic endometrial tissue of Group 3; Group 3B: endometriotic tissue of Group 3 (all images × 400, hematoxylin and eosin).

**Figure 2:**
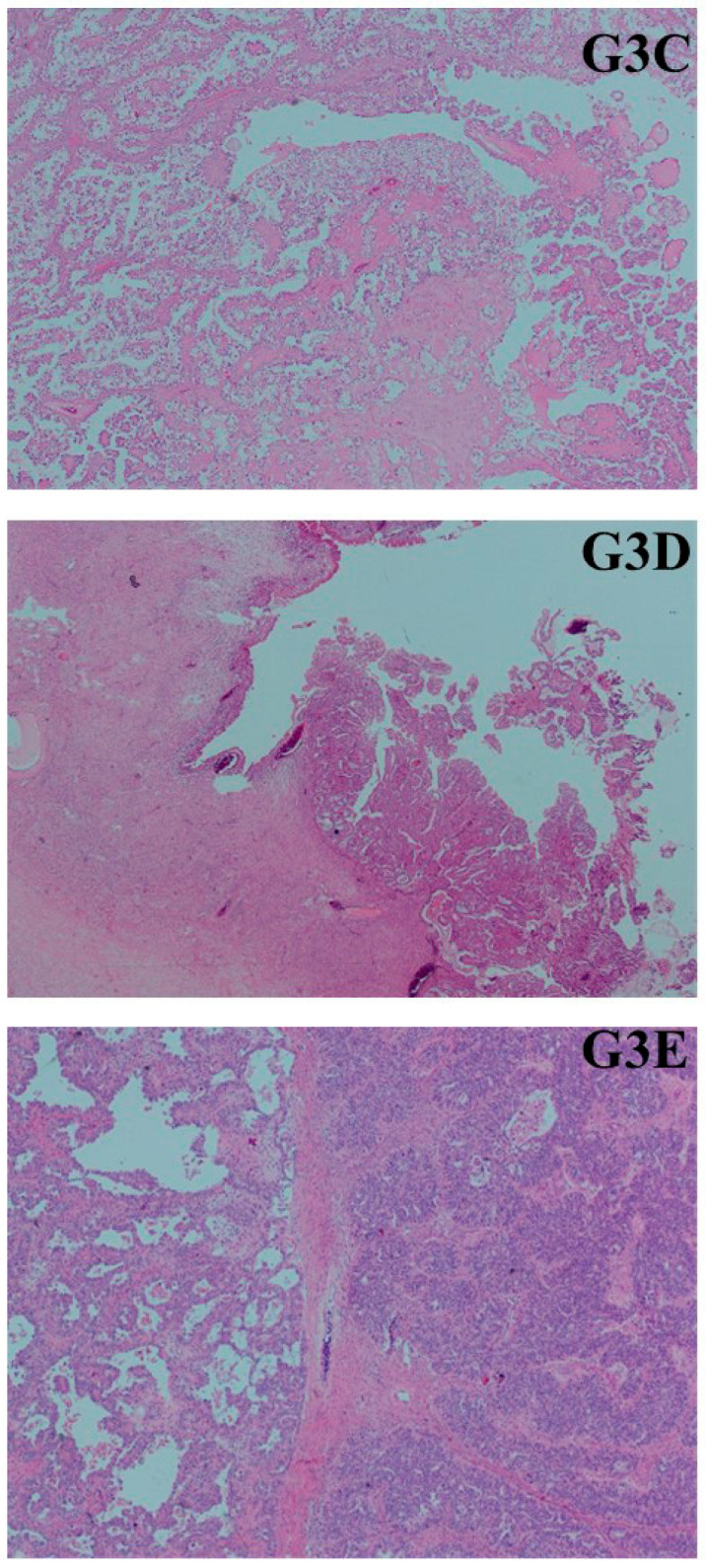
Group 3: Ovarian cancer tissues associated with endometriosis in Group 3. G3C: clear cell carcinoma; G3D: endometrioid-type cancer; G3E: high-grade serous carcinoma (all images × 400, hematoxylin and eosin).

This study consisted of a total of 33 patients and 69 paraffin block tissue samples from these patients, with 10 women in Group 1 (10 eutopic endometrial tissue samples), 10 women in Group 2 (10 eutopic endometrial tissue samples + 10 endometriosis tissue samples), and 13 women in Group 3 (13 eutopic endometrial tissue samples + 13 endometriosis tissue samples + 13 ovarian cancer tissue samples) (Figure 1 and Figure 2). The miRNA levels in these groups were examined through real-time PCR analysis of the paraffin block tissue samples (Table 1 and Supplementary Materials). The average age of the patients was 54 in Group 1 (median age: 51, age range: 40–74), 43.6 in Group 2 (median age: 43, age range: 39–49), and 50.5 in Group 3 (median age: 51, age range: 37–78). In the histopathological examination of the 13 ovarian carcinomas in Group 3, six tumors were clear cell carcinoma (46.2%), four tumors were endometrioid carcinoma (30.8%), and three tumors were high-grade serous carcinoma (23.1%; HGSC). All tumors originated from the tubo-ovarian region.

### 3.2. RT-PCR miRNA 21 and miRNA 200b Analysis Findings

A significant difference was detected in terms of both miRNA expression levels between eutopic endometrium tissues in Group 1 and Group 3 (*p value for miRNA 21: 0.010, p value for miRNA 200b: 0.036*). Accordingly, miRNA 21 and miRNA 200b expression levels were higher in the eutopic endometrium of cancer cases in Group 3.

A significant difference was detected in the expression levels of both miRNAs between eutopic endometrium tissues in Group 1 and Group 2 (*p value for miRNA 21: 0.011, p value for miRNA 200b: 0.007*). Accordingly, miRNA 21 and miRNA 200b expression levels were higher in Group 1 eutopic endometrium.

A significant difference was detected between eutopic endometrium tissues in Group 2 and Group 3 in terms of both miRNA expressions (*p value for miRNA 21: 0.0001, p value for miRNA 200b: 0.002*). Accordingly, miRNA 21 and miRNA200b expression levels were higher in the eutopic endometrium of cancer cases in Group 3.

When eutopic endometrium and foci of endometriosis in Group 2 were compared, a significant difference was found in terms of both miRNA expression levels (*p value for miRNA 21: 0.001, p value for miRNA 200b: 0.003*). In Group 2, miRNA 21 and miRNA 200b expression levels were higher in foci of endometriosis than in eutopic endometrial tissue.

No significant difference was found in terms of miRNA expression levels between the eutopic endometrium and endometriosis foci of cancer cases in Group 3 (*p* value for miRNA 21: 0.648, *p* value for miRNA 200b: 0.376). No significant difference was found between eutopic endometrium and cancer foci (*p* value for miRNA 21: 0.650, *p* value for miRNA 200b: 0.728). No significant difference was found between endometriosis and cancer foci (*p* value for miRNA 21: 0.582, *p* value for miRNA 200b: 0.228).

A significant difference in miRNA expression levels between eutopic endometrium in Group 1 and endometriosis in Group 2 was detected only for miRNA 200b (*p* value for miRNA 21: 0.133, *p value for miRNA 200b: 0.001*). The expression level of miRNA 200b in foci of endometriosis in Group 2 was higher than that of eutopic endometrium tissue in Group 1.

A significant difference was detected in the expression levels of both miRNAs between the eutopic endometrium in Group 1 and the endometriotic foci in Group 3 *(p valuefor miRNA 21: 0.029, p value for miRNA 200b: 0.023)*. The expression levels of miRNA 21 and miRNA 200b in the endometriotic foci in Group 3 were higher than in the eutopic endometrial tissue in Group 1.

A significant difference between foci of endometriosis in Group 2 and Group 3 was detected only for miRNA 200b (*p value for miRNA 200b: 0.010*, *p* value for miRNA 21: 0.230). The miRNA 200b level was higher in foci of endometriosis in Group 2 cases.

**Figure 3:**
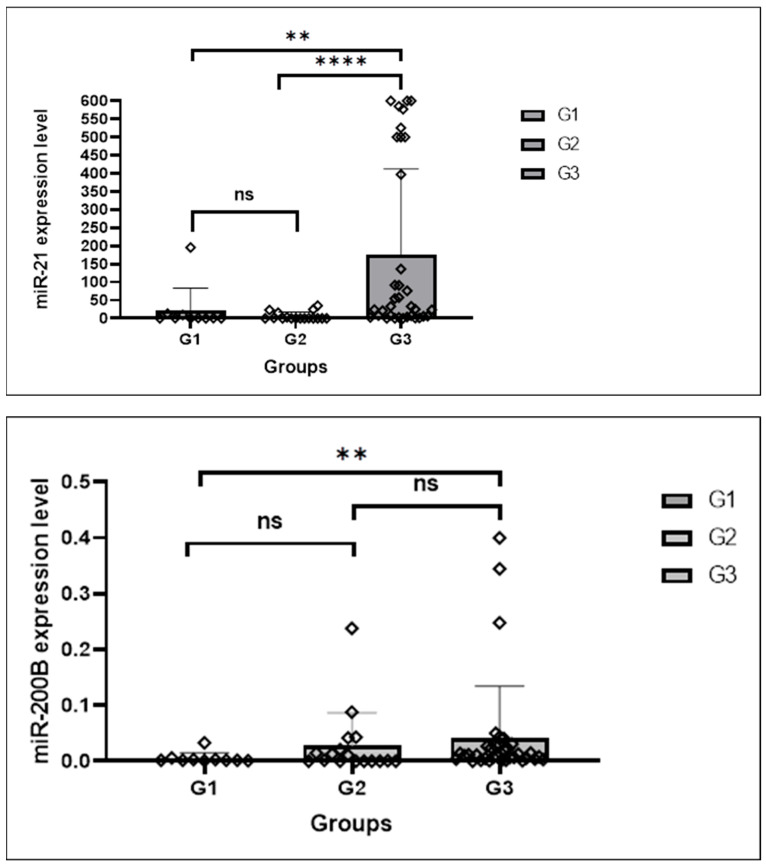
Group 1: eutopic endometrium of cases with no endometriosis; Group 2: Cases with endometriosis; Group 3: Ovarian cancer cases associated with endometriosis. The miR-21 and miR-200b expression levels in groups; ns: not significant; statistical significance *p* < 0.01 **, *p* = 0.0001 ****.

**Figure 4:**
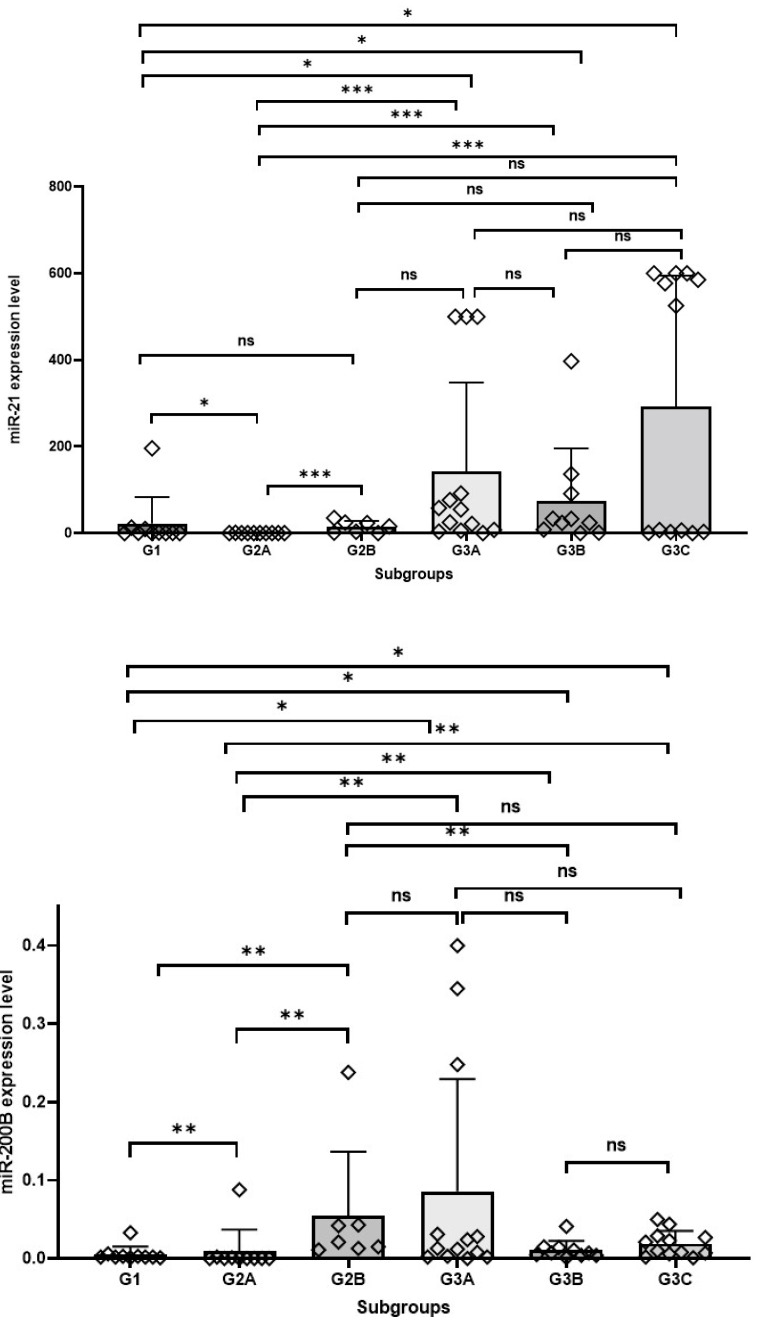
Group 1: eutopic endometrial tissues of Group 1; Group 2A: eutopic endometrial tissues of Group 2; Group 2B: endometriotic tissues of Group 2; Group 3A: eutopic endometrial tissues of Group 3; Group 3B: endometriotic tissues of Group 2; Group 3C: Ovarian cancer tissues of Group 3. The miR-21 and miR-200b expression levels in subgroups; ns: not significant; statistical significance *p* < 0.05 *, *p* < 0.01 **, *p* < 0.001 ***.

A significant difference in miRNA expression levels between cancer tissues in Group 3 and eutopic endometrium in Group 1 was detected for miRNA 200b (*p value for miRNA 200b: 0.014) and* for miRNA 21 (*p* value: 0.025). miRNA 200b and miRNA21 levels were higher in cancer tissue in Group 3 (Figure 3 and Figure 4).

## 4. Discussion

In this study, we found that no significant difference was found in the expression levels of miRNA 21 and miRNA 200b among tissues of eutopic endometrium, endometriosis, and carcinoma foci in EAOC (Group 3). However, the expression levels of miRNA 21 and miRNA 200b in the eutopic endometrial tissue of ovarian cancer cases (Group 3) were significantly higher than those in the eutopic endometrial tissues of cases with (Group 2) and without (Group 1) endometriosis. Additionally, the expression level of miRNA 200b in the endometriotic foci of cases with endometriosis (Group 2) was higher than that in the eutopic endometrial tissue of Group 1 cases.

There are many theories regarding the development of endometriosis. However, none of these theories can solely explain the development of endometriosis when considering the locations of endometriotic foci [1]. Molecular studies have focused mainly on the “retrograde menstruation” and “stem cell origin” hypotheses [9]. Additionally, there are other theories, such as the spread of endometrial cells via hematogenous or lymphatic channels, transformation of peritoneal mesothelial cells, and the development of Müllerian remnants. The retrograde menstruation theory was supported by the identification of ectopic endometrial tissue in the peritoneum during the menstrual cycle, as it is shed via the fallopian tubes. In the stem cell theory, two sites are seen as the reservoirs for stem cells: (i) uterine endometrium (eutopic endometrium) and (ii) cells originating from bone marrow [10]. Under the influence of estrogen, the proliferative phase of adult stem cells contributes to the regeneration of the basal endometrial layer. During the menstrual cycle, the functional layer containing adult stem cells is shed. At this stage, it is accepted that the tissues that undergo retrograde menstruation through the fallopian tubes distribute and localize in ectopic sites. In our study, the lack of significant differences in the expression levels of both miRNAs among eutopic endometrium, endometriosis, and cancer tissues in cases with tubo-ovarian-derived endometriosis and EAOC supports the retrograde menstruation and stem cell theories. The low levels of both miRNA expressions in the eutopic endometrial tissues of the two groups without ovarian cancer further support this finding. This suggests that the real information in determining cases where ovarian cancer may develop on the basis of endometriosis may be hidden in the eutopic endometrium.

Circulating miRNAs have heterogeneous origins, and distinguishing tumor-derived miRNAs from non-tumor-derived miRNAs, such as those from blood cells, can be challenging [1]. However, miRNAs secreted by cancer cells are generally found within extracellular vesicles and exhibit a distinct molecular profile in terms of content and surface markers when compared to those from normal cells. A limiting factor in studying circulating miRNAs is that their expression levels can be influenced by age, gender, ethnicity, and sample type (e.g., whole blood, serum, plasma). Nevertheless, miRNAs are expressed at different levels in normal tissues and, in their neoplastic forms, they are considered reliable diagnostic biomarkers [1]. Therefore, when planning our study consisting entirely of female patients, we first tried to evaluate miRNA 200b and miRNA 21 levels in tissue samples of the same patients, thus minimizing the differences in miRNA levels that may arise from patient age, tissue, and hormonal levels. Subsequently, we demonstrated differences in miRNA expression levels across the groups. In the literature, there are limited numbers of studies investigating changes in miRNA expression levels in human tissues and biological fluids in EAOC (Table 2, [11,12,13,14,15,16]). These studies examined miRNA levels in healthy controls, endometriosis cases, and one or more EAOC subtypes. A common limitation in these studies is that each group consists of a different patient population. miRNAs, beyond PCR, are now being investigated using emerging in situ hybridization techniques to analyze tissue-level miRNA expression [17]. This approach may offer the advantage of assessing tumor morphology as well as miRNA expression under light microscopy.

The miRNA 200 family, which includes miRNA 200a, 200b, 200c, miRNA 141, and miRNA 429, is one of the first families of miRNAs known to exhibit tumor-suppressor effects [8]. A decrease in the levels of this miRNA group promotes tumor progression. Additionally, this group of miRNAs has been associated with cell growth, apoptosis, and epithelial-to-mesenchymal transition in various studies [8]. Among various ovarian cancers, including EAOC, levels of miRNA 200 family members, especially miRNA 200a, 200b, and 200c, have been investigated [14,18,19,20,21]. In the study of Meng et al., a four-miRNA panel (miRNA 200a, miRNA 200b, miRNA 200c, and miRNA 373), which included miRNA 200b, demonstrated a sensitivity of 0.882 and specificity of 0.90 for the diagnosis of EAOC [20]. In another study, miRNA 200b was reported to play a role in the pathogenesis of endometriosis by regulating the stem cell phenotype, proliferation, and invasive extension formation in endometriotic cells by targeting ZEB1, ZEB2, and KLF4 [22]. In our study, the expression level of miRNA 200b in the endometriotic tissues of endometriosis cases (Group 2) was found to be higher than that in the eutopic endometrial tissue of non-endometriosis Group 1 cases. From this finding, we hypothesize that miRNA 200b could be a biomarker for endometriosis. Szubert et al. evaluated seven different miRNA levels in tissue samples from 135 patients undergoing surgery for endometriosis and various types of ovarian cancer (EAOC and high-grade serous ovarian carcinoma) [23]. Normal ovarian parenchyma was used as the control tissue. According to their findings, miRNA 200b expression was lower in ovarian cancer tissue compared to normal ovarian parenchyma and endometriotic foci. However, miRNA 200b levels did not differentiate histological subtypes of ovarian cancer [23].

miRNA 200b, as part of miRNA panels (especially with miRNA 200a and miRNA 200c), has been proposed as a diagnostic biomarker for ovarian cancer in clinical applications due to its high specificity and sensitivity, as demonstrated in various meta-analyses and in-silico-based studies [8]. Furthermore, miRNA 200b has been identified as a prognostic marker indicating poor survival in EAOC in some studies [19,20]. Other studies have reported that increased miRNA 200b levels reduce resistance to cisplatin treatment by targeting DNA methyltransferase, thereby inducing cancer cell death [24].

miRNA 21 is one of the most well-known miRNAs, with oncogenic effects and an extensively studied role in carcinogenesis. In epithelial ovarian cancers, increased serum miRNA 21 expression levels have been reported [11,25,26]. In the study of Xu et al., serum miRNA 21 levels were found to be higher in epithelial ovarian cancers compared to healthy control groups. Increased serum miRNA 21 levels were associated with shorter overall survival, advanced FIGO stage, and high tumor grade. Thus, due to its diagnostic and prognostic value, miRNA 21 has been proposed as a biomarker for epithelial ovarian cancer [27]. Other studies have linked miRNA 21 expression to negative regulation of PTEN and resistance to cisplatin treatment [25,28]. However, no specific finding between tissue levels of miRNA 21 and EAOC has been reported in the literature, to the best of our knowledge. In our study, in cases with EAOC, we did not find a significant difference in the expression levels of miRNA 21 and miRNA 200b among the eutopic endometrium, endometriosis, and cancer foci. However, miRNA 21 and miRNA 200b levels in the eutopic endometrial tissue of cases with EAOC were higher than in eutopic endometrial tissues of both groups of non-cancer cases. This finding suggests that the increased expression of miRNA 21 and 200b in eutopic endometrium may assist in identifying cases at risk of ovarian cancer in the context of endometriosis.

Studies on the development of miRNA-based biosensors for use in early detection of diseases are currently being conducted [8]. Since ovarian cancers are typically diagnosed in later stages, studies that contribute to early diagnosis are of great importance. Various biosensor types, particularly those containing miRNA 21, have been identified for the early diagnosis of epithelial ovarian cancer, either alone or in panels, using serum or tissue levels [8,29]. Biosensors are rapidly developing devices that are used to detect miRNA levels in cancer diagnosis and provide quick, reliable results [8].

The limitations of our study include its retrospective design, small sample size, and the limited number of miRNAs investigated. However, in this retrospective study, we aimed to compare miRNA expression levels in tissue samples from the same patients when designing the groups. This allowed us to show the differences in miRNA expression levels among eutopic endometrium, endometriosis, and cancer tissues. We selected two miRNAs for this study. miRNA 200b was chosen because its effects in relation to our research topic have been demonstrated in previous studies and in silico analyses. miRNA 21 was selected due to its oncogenic effects in many solid organ tumors, including epithelial ovarian cancers and because biosensors targeting miRNA 21 have been developed. Given that ovarian cancers are typically diagnosed in advanced stages and have an aggressive course, early diagnosis is crucial.

## 5. Conclusions

To the best of our knowledge, this is the first study in the literature to compare miRNA levels in the eutopic endometrium, ectopic endometrium, and ovarian cancer tissues from the same individuals. Accordingly, miRNA 200b and miRNA 21 levels were found to be higher in the eutopic endometrial tissue of EAOC cases compared to non-cancer cases. This suggests that increased miRNA 200b and miRNA 21 expression levels detected in eutopic endometrial tissues in cases with endometriosis may contribute to identifying cases that may develop EAOC.

## Figures and Tables

**Table 1:** The properties and miRNA expressions (fold change) of patients in groups.

Groups	Mean Age	Tissue	Mean miR-21 Expression (Fold Change)	Mean miR-200b Expression (Fold Change)
Group 1	54	Eutopic endometrium	22.19	0.005
Group 2	43.6	Eutopic endometrium	0.18	0.009
		Endometriosis	14.77	0.055
Group 3	50.5	Eutopic endometrium	141.84	0.086
		Endometriosis	74.84	0.011
		Cancer foci	292.32	0.019

**Table 2:** miRNA Levels in Serum/Plasma, Tissue, or Ascitic Fluid in Epithelial Ovarian Cancer Cases.

Author, Reference No.	Material	Number of Cases, Material	miRNA Levels Detected in Cancer Tissue
Suryavanshi, S., 2013 [11]	Plasma	14 EOC, 33 Endometriosis, 20 Healthy Controls	Increased levels: miR-15b, miR-16, miR-21, miR-195
Dong, M., 2015 [12]	Serum and Tissue	12 EOC, 12 Endometriosis, 12 Healthy Controls	Increased levels: miR-191
Tian, X., 2015 [13]	Tissue	10 EOC, 10 Ovarian Endometrioma, 10 Healthy Controls	Increased levels: miR-191
Braicu, O.L., 2017 [14]	Tissue	8 EOC (Endometrioid Ca), 9 Endometriosis, 5 Healthy Control Ovarian Tissues	Decreased levels: miR-200b
Nakamura, N., 2020 [15]	Serum and Ascitic Fluid	7 EOC (4 Endometrioid Ca, 3 Clear Cell Ca), 34 Ovarian Endometrioma	Increased levels: miR-486-5p
Kumari, P., 2021 [16]	Tissue	10 Endometriosis, 10 Endometrioid-Type Ovarian Ca, 10 Healthy Control Tissues	Increased levels: miR-99b, miR-125a, miR-143, miR-145 Decreased levels: miR-16, miR-20a

## Data Availability

The original contributions presented in this study are included in the article/Supplementary Materials. Further inquiries can be directed to the corresponding author.

## Supplementary Materials

The following supporting information can be downloaded at: https://www.mdpi.com/article/10.3390/medicina61061035/s1, Table S1: RT-PCR analysisresults of thecases.
