# Supplementary material for: The Effect of MicroRNA 21 and MicroRNA 200b Expression on Carcinogenesis in Endometriosis-Associated Ovarian Cancers and Relationship with Clinicopathological Parameters
Source: Medicina (Kaunas). 2025 Jun 4;61(6):1035. doi: 10.3390/medicina61061035 (PMC12195162; doi:10.3390/medicina61061035)
Supplement: Supplementary file 1 [file medicina-61-01035-s001.zip › medicina-3627358-supplementary.pdf]

medicina

MDPI

Supp. Table S1. RT-PCR analysisresults of thecases

Groups	Age	Histopathologicalevaluation (Diagnosis)	mir-21	mir-200b
1	54	eutopicendometrium	0,773	0,001
1	69	eutopicendometrium	0,588	0,033
1	74	eutopicendometrium	0,111	0,006
1	48	eutopicendometrium	1,133	0,002
1	48	eutopicendometrium	13,002	0,003
1	47	eutopicendometrium	0,252	0,003
1	49	eutopicendometrium	195,971	0,002
1	58	eutopicendometrium	0,254	0,002
1	53	eutopicendometrium	0,028	0,001
1	40	eutopicendometrium	9,771	0,002
2	47	eutopicendometrium	0,102	0,088
2	45	eutopicendometrium	0,123	0
2	42	eutopicendometrium	0,122	0
2	44	eutopicendometrium	0,08	0,002
2	41	eutopicendometrium	0,146	0
2	41	eutopicendometrium	0,241	0
2	39	eutopicendometrium	0,047	0
2	49	eutopicendometrium	0,958	0,001
2	48	eutopicendometrium	0,009	0
2	40	eutopicendometrium	0,014	0
2	47	endometriosis	0,144	0,015
2	45	endometriosis	23,388	0,042
2	42	endometriosis	1,065	0,013
2	44	endometriosis	24,602	0,238
2	41	endometriosis	15,781	0,043
2	41	endometriosis	35,013	0,021

2	39	endometriosis	3,372	0,011
2	49	endometriosis	NO	NO
2	48	endometriosis	NO	NO
2	40	endometriosis	NO	NO
3	48	eutopicendometrium	0,028	0
3	49	eutopicendometrium	76,278	0,248
3	52	eutopicendometrium	25,026	0,028
3	52	eutopicendometrium	7,599	0,013
3	46	eutopicendometrium	91,578	0,003
3	52	eutopicendometrium	57,939	0,031
3	51	eutopicendometrium	640,722	0,345
3	39	eutopicendometrium	577,104	0,012
3	56	eutopicendometrium	3,632	0,002
3	78	eutopicendometrium	1288,13	0,544
3	37	eutopicendometrium	21,197	0,024
3	53	eutopicendometrium	5,885	0,002
3	44	eutopicendometrium	54,793	0,008
3	48	endometriosis	0,038	0,001
3	49	endometriosis	91,103	0,011
3	52	endometriosis	8,343	0,005
3	52	endometriosis	397,011	0,004
3	46	endometriosis	136,171	0,007
3	52	endometriosis	23,863	0,041
3	51	endometriosis	NO	NO
3	39	endometriosis	33	0,015
3	56	endometriosis	24,337	0,014
3	78	endometriosis	NO	NO
3	37	endometriosis	NO	NO
3	53	endometriosis	1,158	0,004
3	44	endometriosis	33,382	0,007
3	48	clearcellca	0,877	0,006

3	49	clearcellca	NO	NO
3	52	clearcellca	0,229	0,021
3	52	clearcellca	0,026	0,007
3	46	clearcellca	0,48	0,029
3	52	HGSOC	585,308	0,044
3	51	clearcellca	525,55	0,023
3	39	endometrioidca	5068,62	7
3	56	HGSOC	2,516	0,008
3	78	endometrioidca	2,984	0,01
3	37	endometrioidca	160,117	0,027
3	53	endometrioidca	7,214	0,001
3	44	HGSOC	6,264	0,002
			1	1
			1	1
			1	1
			1	1
			1	1
			1	1

Sample: Coding of tissuesamplesaccordingtogroups, sample ‘E’: eutopictissue; sample ‘H’: endometriosistissue; sample ‘T’: tumortissue, NO: Not obtained, HGSOC: highgradeserousovariancancer, STD: standart
